# Structural and Chemical Changes in Si Nanoparticle-Based Anodes in Lithium-Ion Batteries during the (De)lithiation Processes Studied by In Situ Raman Spectroelectrochemistry

**DOI:** 10.1021/acsaem.5c00066

**Published:** 2025-04-28

**Authors:** Zuzana Vlčková Živcová, Farjana J. Sonia, Martin Jindra, Martin Müller, Jiří Červenka, Antonín Fejfar, Otakar Frank

**Affiliations:** † J. Heyrovský Institute of Physical Chemistry, Czech Academy of Sciences, 182 23 Prague, Czech Republic; ‡ Department of Physical Chemistry, University of Chemistry and Technology, 16628 Prague, Czech Republic; § Institute of Physics of the Czech Academy of Sciences, 182 21 Prague, Czech Republic

**Keywords:** silicon nanoparticles, in situ Raman spectroelectrochemistry, Li-ion battery, electrochemistry, SEI layer

## Abstract

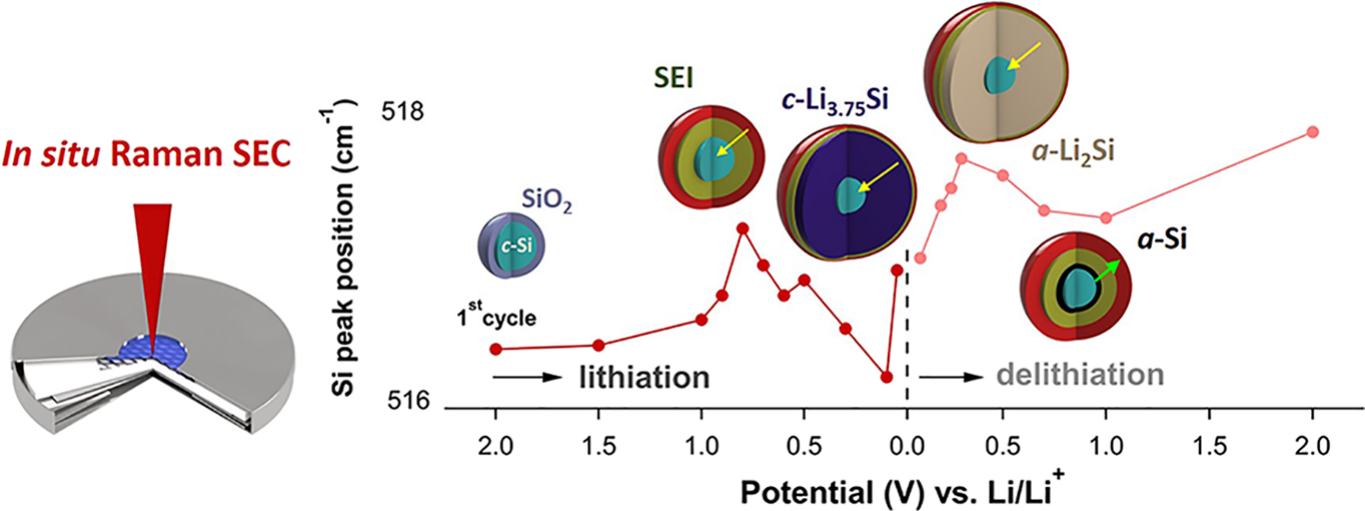

Nanostructured silicon is considered one of the most attractive anode materials for high-energy-density Li-ion batteries (LIBs) because it can provide a high capacity and extended cycle life compared to bulk Si anodes. However, little is known about the electrochemical lithiation mechanism in nanosilicon due to the lack of suitable measurement techniques. In this study, nanostructured anodes based on Si nanoparticles (approximately 6 nm) integrated within a conductive carbon-based matrix are studied by an in situ Raman spectroelectrochemical (SEC) method in modified coin cells in LIBs. Additionally, cyclic voltammetry and galvanostatic charge–discharge cycling are used to determine the stability of the solid electrolyte interphase (SEI) layer and the long-term capacity degradation of the Si nanoparticle-based anodes. The in situ Raman SEC provides unique insight into the crystal lattice changes and degradation/amorphization pathways of the Si nanocrystals and the electrolyte (LiPF_6_ in EC/DMC) decomposition during the electrochemical lithiation and delithiation processes. The evolution of the spectral parameters (shift, line width, intensity) of the first-order Raman peak of crystalline Si at 520 cm^–1^ is found to be related to the stress buildup in the nanoparticles. This stress originates from the (i) SEI layer formation on the electrode surface within the initial charge/discharge cycle, (ii) the lithiation-induced stress in Si nanoparticles and the native oxide on their surface, and also (iii) the progressive crystalline-to-amorphous Si phase transition. The structural changes in the anodes determined using in situ Raman SEC show good agreement with the results obtained from cyclic voltammetry measurements, revealing a progressive crystalline-to-amorphous Si phase transition and a complex energy storage mechanism in nanostructured silicon anodes in LIBs.

## 1 Introduction

Silicon anodes have long been considered one of the most promising anode materials for next-generation lithium-ion batteries (LIBs).^1^ Silicon, thanks to the high theoretical specific capacity of 3579 mAh/g (for Li_3.75_Si, the highest lithiated crystalline phase achievable for ambient temperature lithiation), has the potential to replace the most commonly used graphite with a ca. ten times lower specific capacity (372 mAh/g for LiC_6_).^2^ However, since silicon is an electrically nonconductive material, it is still necessary to use some conductive matrix, e.g., carbon, as in the form of silicon/carbon composite anodes.^3^ The application of Si anodes has thus far been limited, mainly due to a significant volume expansion of 280% upon full lithiation of Si^4^ and the related tensile/compressive stresses arising during lithiation/delithiation cycles. These issues have negatively affected the Si anode capacity and stability in LIBs, leading to cracking and pulverization of the Si anodes during long-term cycling.^5^ The solution to overcome this disadvantage (lithiation-induced stress) is to replace bulk silicon with nanostructured (e.g., nanoparticles, nanowires) silicon anodes. By increasing the surface area-to-volume ratio of Si, (i) the internal strain during lithiation is reduced, (ii) the accessibility for the electrolyte increases, (iii) the volumetric energy density of the composite anode decreases, and (iv) the amount of native surface SiO_2_ increases.^6^ Moreover, nanosizing of the electrode materials also promises higher (dis)charge rates because it shortens the length of the rate-limiting diffusion pathway of Li-ions and electrons through the electrode material.^7^ Silicon nanoparticles (SiNPs) naturally contain SiO_*x*_ on the surface, which influences the SiNPs’ electrochemical properties.^6,8−10^ This silicon oxide layer is additionally composed of hydroxylated termination groups (SiOH), which increases the surface reactivity.^11^ The utilization of nano-Si has largely addressed the active material pulverization issue because nanoparticles can better accommodate the large strains associated with lithiation.^12,13^ Liu et al. studied the lithiation of individual silicon nanoparticles in real time with in situ transmission electron microscopy (TEM).^14^ They established a critical Si particle diameter of ∼150 nm, below which the particles neither cracked nor fractured upon first lithiation, whereas particles above this size formed surface cracks and then fractured due to lithiation-induced swelling. An important observation was made by Sethuraman et al.,^15^ who measured stress induced by (de)lithiation of Si thin film on an incompressible substrate, where the stresses in the Si anode directly influenced its capacity and energy dissipation due to plastic deformation.

The proper understanding of the growth mechanism and cycling stability of the solid electrolyte interphase (SEI) layer emerging on the silicon electrode surface during electrochemical reactions of salts and organic-solvent-based electrolytes is another crucial factor in the ability to control the LIBs' properties. Various models of the SEI layer formation have been proposed over the years.^16,17^ The mosaic structure model assumes that the SEI layer is composed of a mixture of inorganic and organic components.^18^ This model suggests that the SEI layer is not a uniform structure but a mosaic-like arrangement of different compounds, where the contribution of the conduction of Li^+^ at the grain boundaries cannot be neglected. The inorganic components provide stability and passivation, while the organic components contribute to the formation of a porous structure. Another model describes the SEI layer as a bilayer structure. The inner (bottom) layer is formed by inorganic components, such as LiF, Li_2_O, Li_2_SiO_3_, LiOH, or Li_2_CO_3_, which are insoluble in the electrolyte, and the outer (top) layer, also known as the organic porous layer, is composed of organic/polymeric (Li-ROCO_2_, C–O–C, etc.) compounds resulting from the decomposition of the electrolyte solvent.^19^ The thickness of SEI is ca. 2–170 nm, depending on the cycling methods or silicon structure.^20^ The flexibility and mechanical stability of the SEI layer are crucial factors for silicon-based anodes and the long-term stability of the battery. The significant irreversible capacity loss within the first discharge of the LIB is attributed to the formation of the SEI layer.^20−23^ Nevertheless, the crucial aspect of the stable SEI layer lies in its ability to prevent further decomposition of the electrolyte. The “breathing” effect, i.e. the gradual change in the composition and thickness of the SEI layer, depending on the discharge and charge cycle, was described in detail by Veith et al.^21,24^ The SEI layer appears to thin (down to 18 nm) as the Si layer swells with increasing Li content (i.e., *c*-Li_15_Si_4_ phase) and thickens (up to 25 nm) upon delithiation.^24^ The stress resulting from volume changes in silicon during lithiation/delithiation could be one potential cause of the mechanical damage of the SEI layer. This layer acts as a barrier to prevent direct contact between the electrode and the electrolyte. It also exhibits ionic conductivity, which allows the transport of lithium ions via a two-layer/two-mechanism diffusion process.^25^

Raman spectroscopy is a very useful nondestructive technique to aid in determining the composition and structure of the material from frequencies and widths of the Raman bands, but also lattice deformation within the material from the relative Raman frequency shifts of these bands. Precisely, deformation refers to a change in atomic positions or chemical bond lengths in a given material resulting from the action of external stress. Compressive and tensile stresses lead to corresponding deformations in the crystal lattice, manifested as shifts of a Raman peak position to higher or lower wavenumbers, respectively. The displacement of the Raman peak is proportional to the magnitude of the applied stress and the ensuing deformation manifested in the lattice.^26−30^ It is important to mention that specific information about the distribution and magnitude of stress cannot be obtained from the Raman spectra, as the Grüneisen parameters for Raman modes of lithiated Si are not yet available. A few in situ Raman spectroelectrochemical studies were performed on silicon nanoparticles and wires before; however, they focused on large nanostructures with a size above 100 nm.^5,31−35^ For instance, Zeng et al.^36^ investigated the lithiation-induced stress in crystalline silicon particles (*c*-Si; 100 nm) using in situ high-pressure Raman measurement. Tardif et al.^35^ integrated operando Raman spectroscopy and XRD to observe the lithiation/delithiation of Si under limited capacity conditions, resulting in the formation of “*c*-Si core/amorphous shell” particles. They fabricated a silicon anode using *c*-Si particles with a wide particle size distribution (20–120 nm) and with the addition of fluoroethylene carbonate (FEC), which influenced the redox potentials of the electrolyte. The solid-state amorphization occurring during silicon lithiation could play a substantial role in stress generation and fracture, ultimately resulting in capacity degradation. Several studies have been conducted using other real-time in situ techniques^37^ to investigate the interfacial processes within silicon anodes. Works dealing with in situ XRD on amorphous Si (*a*-Si)^38^ or crystalline Si (*c*-Si)^39^ studied the phase (Li*_*x*_*Si) formation and changes that occur during the lithiation and delithiation of the Si electrode. Liu et al. used in situ TEM to study the dynamic lithiation process of single-crystal silicon with atomic resolution.^40^ They found out that the lithiation interface between the crystalline silicon (*c*-Si) and the amorphous product of the *a*-Li*_*x*_*Si alloy is atomically sharp with a thickness of ca. 1 nm.^40^ Cao et al. investigated the SEI growth in situ using X-ray reflectivity (XRR). In their earlier works, they proposed a mechanistic atomic-scale three-step lithiation model for crystalline silicon where the inorganic SEI layer exhibits breathing behavior during lithiation (SEI thickness increases) and delithiation (decreases).^20,41^ The later work^42^ on native oxide-terminated silicon wafers provides novel mechanistic insights into the SEI growth process on Si, showing the formation of two well-defined sublayers within the inorganic SEI layer referred to as Si-SEI junction (containing mostly Li_*x*_SiO_*y*_ and Li_*x*_Si_*y*_) and SEI-electrolyte interface (LiF).

In this work, to the best of our knowledge, we present the first in situ Raman spectroelectrochemical (SEC) study monitoring the real-time potential-dependent structural changes in ultrasmall silicon nanoparticles, focusing on the investigation of the stress evolution in *c*-Si, and SEI layer growth and stability in composite silicon nanoparticle-based anodes. The anodes, prepared with SiNPs embedded in a conductive carbon matrix, were tested in a half-coin cell configuration, focusing on the first and second lithiation/delithiation cycles. The noncommercial synthesized ultrasmall SiNPs used in our work, compared to the larger SiNPs from commercial sources used in other in situ Raman studies, have a very narrow, uniform particle size distribution with a size of ca. 6 nm. Furthermore, the formation of thinner outer layers on our SiNPs during (de)lithiation allows observation of stress evolution in the unreacted *c*-Si core using in situ Raman SEC. Based on these observations, we propose a qualitative model of the *c*-Si core changes as a dependence of the applied (de)lithiation potential, which provides unique insight into the fundamental energy storage mechanisms in nano-Si-based anodes in LIBs. Also, we correlate these findings with the cyclic voltammetry measurements.

## 2 Experimental Section

### 2.1 Materials and Electrode Preparation

**Figure 1:**
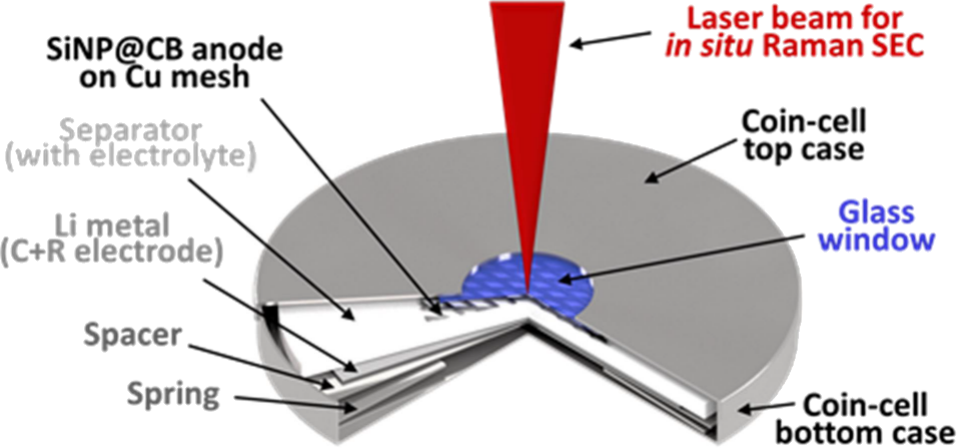
Schematic picture of a coin half-cell battery for in situ Raman spectroelectrochemical measurements.

The silicon nanoparticles (SiNPs) were prepared by plasma-enhanced chemical vapor deposition (PECVD) in a continuous flow-through glass tube reactor with an RF silane/argon nonthermal plasma at 90 W power, according to our previous work.^43^ The as-grown Si particles (approximately 6 nm) were transferred to the glovebox, and the whole process of electrode preparation was carried out inside the glovebox to prevent oxidation of the particles. For preparing the silicon/carbon electrodes (SiNP@CB), the SiNPs were mixed with the conductive carbon black (Super-P, Imerys, Paris, France) and polyvinylidene difluoride (PVDF, Kynar HSV 1800, Arkema, Colombes, France) binder in a 60:20:20 wt ratio using a mortar and pestle. *N*-methyl-2-pyrrolidone (NMP, ≥99.8%, Roth, Karlsruhe, Germany) was further added (200 μL) as an organic solvent to the mixture of dry powders to prepare a slurry of optimum consistency and tape-casted on Cu foil current collector (for investigating electrochemical behavior) and Cu mesh (for in situ Raman SEC studies). The coated films were subsequently dried on a hot plate at 80 °C for 6 h. The as-prepared noncalendered electrodes (working electrode) were then assembled in CR2032 coin-cells as a half-cell configuration (Figure 1 cell with glass window for in situ Raman SEC) using Li-metal as a counter and reference electrode. The electrolyte was a solution of 1 M LiPF_6_ in EC/DMC (50:50 vol ratio, Sigma-Aldrich, product no. 746711).

### 2.2 Characterization Methods

The SiNPs' size and structure were characterized by high-resolution transmission electron microscopy (HR-TEM) using a JEM-2100Plus microscope (accelerating voltage of 200 kV). The cyclic voltammetric measurements were performed at a potential sweep rate of 0.1 mV/s, and the galvanostatic charge/discharge cycles were carried out at a cycling rate of C/10, both in the electrochemical potential window of 0.05–2 V (vs Li/Li^+^) using a μ-Autolab type III galvanostat/potentiostat (Metrohm). In situ Raman spectroelectrochemical (SEC) measurements were performed in a coin cell battery with a glass window (Figure 1) to monitor the structural changes occurring during battery charge/discharge. The Raman spectra were excited using a 633 nm (1.96 eV) He–Ne laser with a power of 1 mW at the sample to avoid the electrode surface degradation, and recorded using a LabRAM HR spectrometer (Horiba Jobin-Yvon) interfaced with an Olympus microscope (objective 50×/0.50). A diffraction grating of 600 lines/mm was used, giving a spectral point-to-point resolution of 1.27 cm^–1^. The spectrometer was calibrated using the *T*_2g_ mode of a bulk Si wafer at 520.2 cm^–1^. The measured Raman spectra were fitted by Lorentzian line shapes. Raman spectra were recorded during the potential-step chronoamperometry at a fixed potential at holding times *t* = 600 and 1200 s after the potential was set (held for long-term current stabilization).

## 3 Results and Discussion

### 3.1 Structural Characterization of Si Nanoparticles

**Figure 2:**
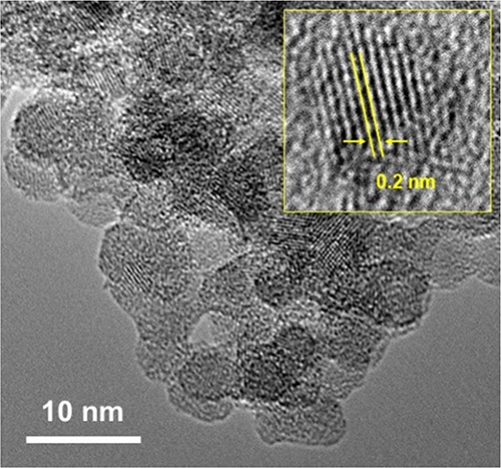
HR-TEM images of individual Si nanocrystals.

Figure 2 shows the HR-TEM images of the SiNPs. The size of particles is approximately 6 nm with a narrow particle size distribution. The surface of the particles is covered by a very thin natural silicon oxide layer of <1 nm, which is smaller than the thickness of the native oxide layer on bulk silicon in the range of 1–4 nm, depending on the oxidation environment and measurement method.^44,45^ The HR-TEM images reveal the presence of lattice fringes with an interplanar spacing of ∼0.2 nm, which corresponds to the (111) plane of crystalline silicon.^46^

### 3.2 Electrochemical Characterization

**Figure 3:**
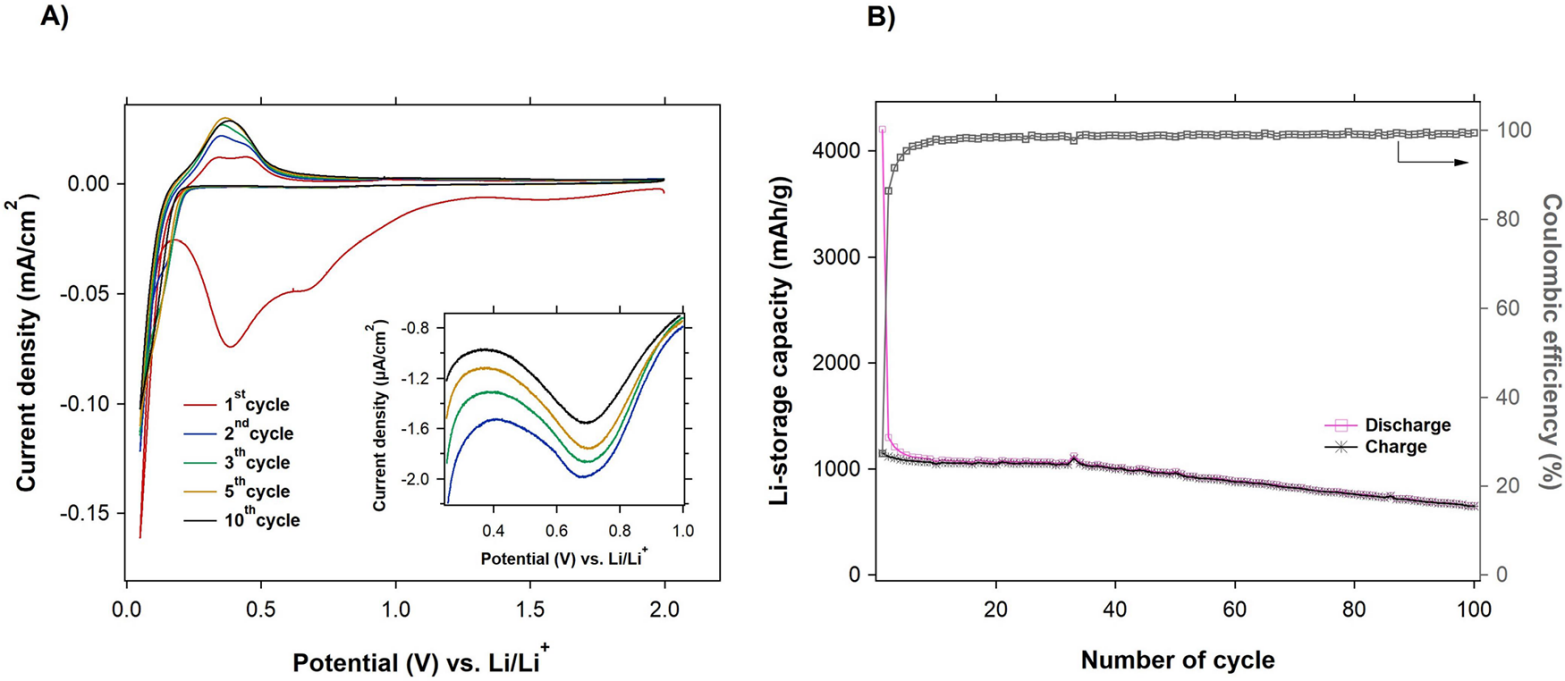
(A) cyclic voltammograms at a scan rate 0.01 mV/s in the range between 0.05 and 2 V vs Li/Li^+^ for the 1st (red line), 2nd (blue line), 3rd (green line), 5th (brown line), and 10th cycle (gray line) and (B) galvanostatic cycling (charge–black line with star symbols, discharge–pink line with square symbols) and Coulombic efficiency (gray line) at a cycling rate C/10 of the SiNP@CB anode.

First, we studied the basic electrochemical cycling performances of battery systems with the SiNP@CB anode using cyclic voltammetry (Figure 3A) and galvanostatic charge/discharge (Figure 3B) methods. Cyclic voltammograms were recorded after the first, second, third, 5th^,^ and 10th cycles at a scan rate of 0.1 mV/s. During the initial lithiation cycle (from 2 to 0.05 V vs Li/Li^+^), a broad reduction peak appears at around 1.5 V. It is reported to be related to the initial decomposition of the LiPF_6_ electrolyte salt, resulting in LiF formation.^47^ As the lithiation continues, the amorphous overlayer consisting of oxides on the Si surface starts to be lithiated at a potential of 1.3 V, which is accompanied by the irreversible formation of Li_2_O and lithium silicates that form a part of the initial SEI layer.^6,9^ Further, two reduction peaks at 0.7 and 0.4 V are visible. They are assigned to the electrolyte solution (LiPF_6_ in EC/DMC) reduction^47−49^ related to the growth of bottom-SEI and the formation of the top-SEI, respectively.^42^ In the second and subsequent cycles, the large reduction peak corresponding to the growth of the top-SEI layer disappears completely, while the reduction peak related to the bottom-SEI persists but with a much lower and continuously decreasing current (see Figure 3A inset). The lithiation of crystalline Si (*c*-Si) is reflected in the presence of reduction peaks in the range of ca. 0.2–0.1 V. They are related to the formation of amorphous phases, *a*-Li*_*x*_*Si, with *x* increasing from 2 (*a*-Li_2_Si, at ca. 0.2 V) up to 3.5 in the highly lithiated phase *a*-Li_3.5_Si (at ca. 0.1 V).^39,50,51^ The lithiated amorphous phases still coexist with the initial nonlithiated *c*-Si. The *a*-Li_3.5_Si phase further rapidly crystallizes in the range of 0.08–0.05 V, depending on the Si particle size, into the fully lithiated crystalline phase *c*-Li_3.75_Si.^50^ The subsequent delithiation process (from 0.05 to 2 V vs Li/Li^+^) causes the *c*-Li_3.75_Si phase to be reverted to Li*_*x*_*Si phases represented by the two oxidation peaks at 0.32 V (*a*-Li_3.5_Si to *a*-Li_2_Si) and 0.45 V (*a*-Li_2_Si to *a*-Si).^52^

Figure 3B illustrates the capacity retention during 100 galvanostatic charge–discharge cycles at a rate of C/10. The SiNP@CB electrode provides the initial discharge capacity of 4203 mAh/g with irreversible loss, making the value of 1296 mAh/g during the first charge (Coulombic efficiency ∼ 31%), caused by the initial growth of the SEI layer. Furthermore, the PVDF binder creates a dense and conformal coating on the silicon particles, which may trap lithium ions after the first lithiation cycle, causing irreversible capacity loss.^53^ Even though the first discharge specific capacity reaches the value of the theoretical capacity corresponding to the *c*-Li_4.4_Si phase (4200 mAh/g), the formation of the *c*-Li_3.75_Si phase with a theoretical capacity of 3579 mAh/g^54^ is much more probable in the room temperature electrochemical lithiation process and is accompanied by the side reactions connected with the SEI layer formation, which increase the first-cycle specific capacity beyond the theoretical limit. After 100 cycles, the Coulombic efficiency reaches close to 100%, and the reversible (i.e., charge) capacity is approximately 650 mAh/g.

### 3.3 In Situ Raman Spectroelectrochemistry

**Figure 4:**
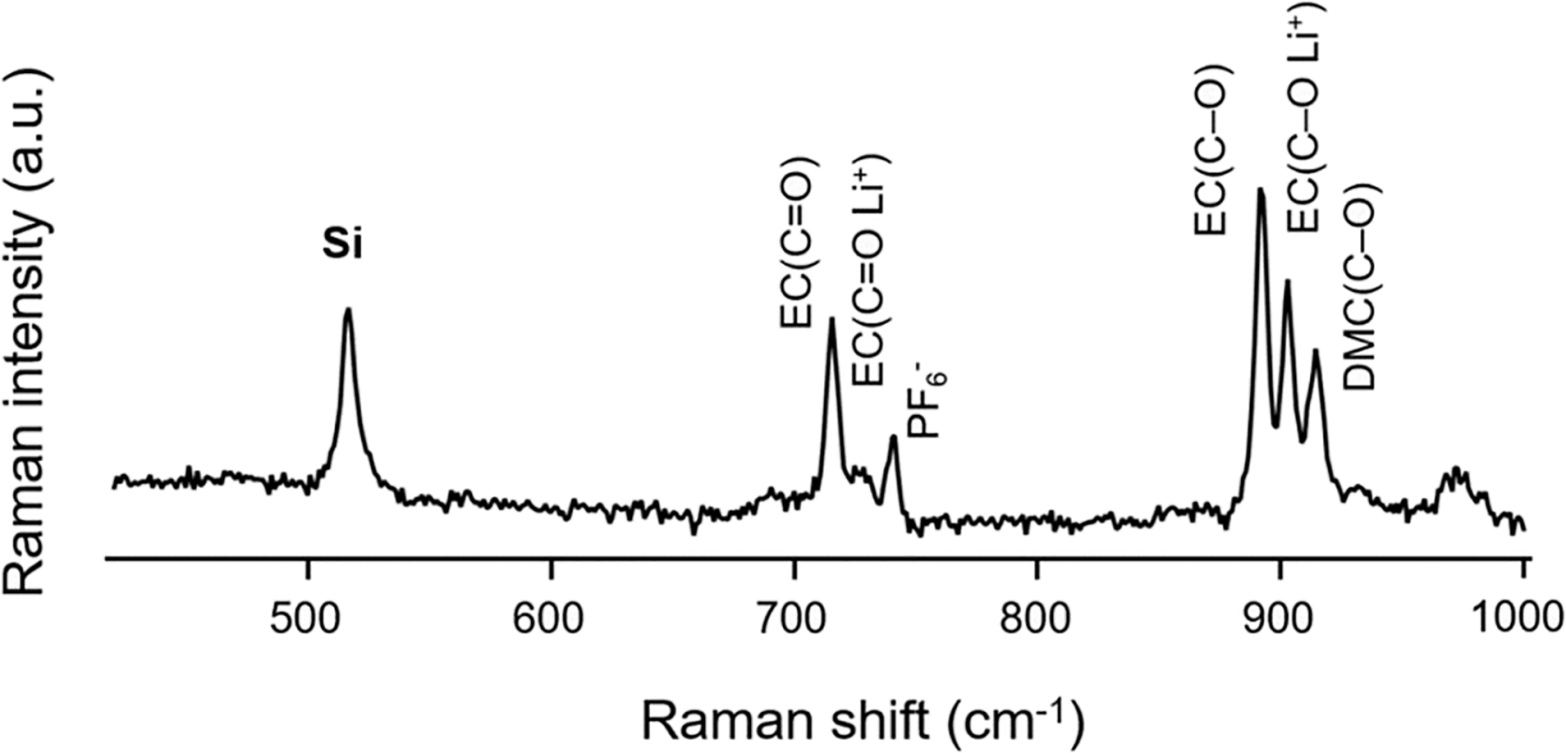
Raman spectrum of the pristine SiNP@CB electrode before cycling with the spectral regions of interest. The first (from the left to right) is related to the Si Raman band (520 cm^–1^), and the other two, at 700–760 and 870–940 cm^–1^, correspond to the Raman bands of the electrolyte solution (LiPF_6_ in EC/DMC).

In situ Raman SEC provides real-time information (within the time resolution provided by the minimum acquisition time needed to capture spectra with a high enough signal-to-noise ratio) on the crystal lattice changes (stress analysis) and possible degradation or amorphization processes of SiNPs during charge/discharge cycles but is also used to study the electrolyte decomposition connected with SEI layer formation. There are two spectral regions of interest: (i) the region of the first–order zone-center unstrained crystalline silicon (*c*-Si) Raman peak at ∼520 cm^–1^, and (ii) Raman peaks related to LiPF_6_ in EC/DMC electrolyte solution in the range of 700–950 cm^–1^ (Figure 4). Before acquiring the Raman spectra, we used chronoamperometric charging at selected potential steps with a 600 s holding time and repeated the measurement after an additional 600 s to interrogate the influence of the reaction time on the spectral response. We note that these holding times result in slower (dis)charging than in most other studies^31−35^ and can be the reason for some of the differences reported below. The stability of the optical system during long-term Raman measurements (without applied voltage) before in situ Raman SEC is displayed in Figure 1 (Supporting Information). The silicon anode exhibited high stability over 40 min, with the Si Raman peak remaining at a constant position at 517.6 ± 0.1 cm^–1^. Subsequently, the battery was connected to the circuit, and in situ Raman SEC measurements were performed.

**Figure 5:**
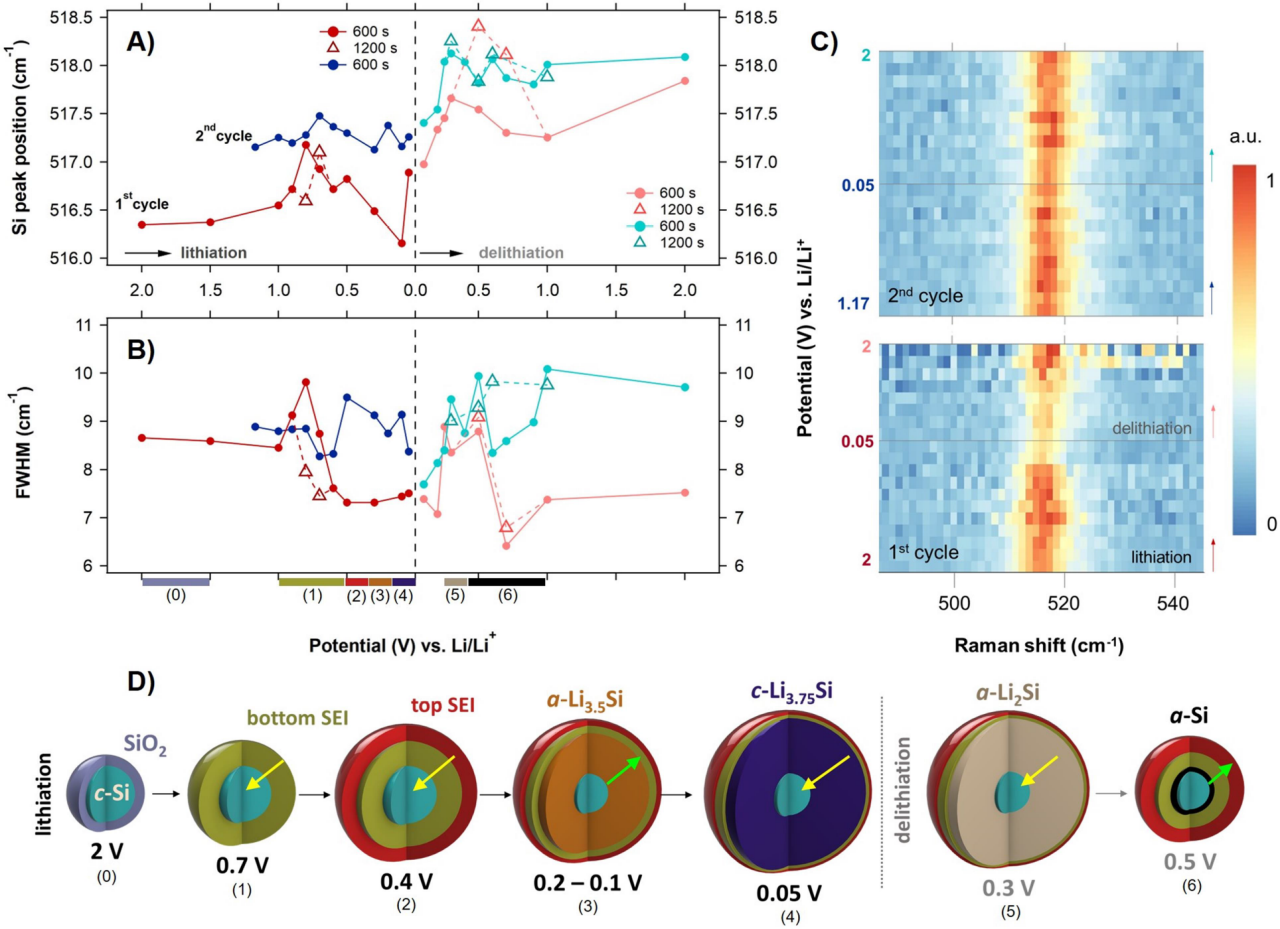
(A) Si Raman peak position as a function of applied potential (vs Li/Li^+^), and (B) FWHM determined from Lorentzian fits of the Raman spectra (Figure S2) of SiNP@CB electrode during lithiation (left part of the charts) and delithiation (right part of charts) within the 1st (dark/light red lines) and 2nd (dark/light blue lines) cycle. All spectra are recorded at the holding time *t* = 600 s (full lines with circles), and, additionally, for selected potentials (lithiation; 1st cycle at 0.7 and 0.8 V, delithiation; 1st cycle at 0.5 and 0.7 V, 2nd cycle at 0.3, 0.5, 0.6, and 1 V), also at *t* = 1200 s (dashed lines with triangles). (C) Raman intensity map of the Si Raman band (520 cm^–1^) as a function of the applied potential (vs Li/Li^+^; shown on the left side); *t* = 600 s. (D) Proposed model of *c*-Si core (light blue) compression (yellow arrow)/tension (green arrow) during the first (de)lithiation based on in situ Raman SEC measurement. The individual steps (1–6) labeled below graphs (A and B) on the *X*-axis using colors representing the specific SEI layers (1–olive, 2–red), Li*_*x*_*Si phases (3–dark amber, 4–indigo, 5–light brown), and *a*-Si (6–black) align with the potential regions of the respective transformation.

A series of in situ Raman spectra of the SiNP@CB anode as a function of the applied potential during the first (dark and light red spectra; lithiation in the range from 2 to 0.05 V, delithiation from 0.05 to 2 V vs Li/Li^+^) and the second (dark and light blue spectra; lithiation in the range from OCP potential at 1.17 to 0.05 V, delithiation from 0.05 to 2 V vs Li/Li^+^) cycle in the spectral range of 200–1000 cm^–1^ is shown in Figure S2. All of the peaks were fitted using Lorentzian functions (Figures S3A and S4). The shifts and full-width half maxima (FWHM; Figure 5A,B), as well as the intensity changes (Figures 5C and S3B) of the *c*-Si Raman band, were determined during the first two charge/discharge cycles. The map of Raman intensity normalized to the Raman spectrum of the pristine electrode before cycling (Figure 4) in Figure 5C shows the evolution of the Si Raman band as a function of the applied potential. The results show that the intensity variations of the *c*-Si Raman peak during the first and second cycles are different. In the first lithiation cycle, the Si peak intensity is relatively stable until the onset of lithiation of *c*-Si at 0.3 V, after which it rapidly decreases. The lowered intensity remains until the delithiation takes place again at 0.3 V and is reverted to the almost initial value at 0.7 V. Only a minor further increase is observed until the cycle’s end. In the second cycle, the Si peak intensity is more stable, and only a small drop is visible at the lowest potentials. Other trends are shadowed by fluctuations caused, e.g., by differences in laser focus. The first cycle intensity decrease of the bands corresponding to EC/DMC (C=O and C–O vibrational modes^55−57^) in the spectral region of 700–1000 cm^–1^ during lithiation at reduction potentials up to 0.8 V might indicate the electrolyte decomposition connected with the growth of SEI layers. However, we did not detect the SEI-forming products (primarily LiF and Li_2_O with Raman modes around 300–400 and 530 cm^–1^, respectively^58,59^) in our in situ Raman spectra (Figure S2). Nevertheless, their low Raman intensity does not imply their absence from the SEI layer. Within the second cycle, the intensities of these Raman bands are significantly more stable, showing stabilization of SEI layers (Figure S4).

Based on the dependency of the *c*-Si peak shift (Figure 5A) on the applied potential, i.e., on the lithiation/delithiation, determined from Lorentzian fitting, we constructed a model (Figure 5D) of *c*-Si core compression/tension during the first (de)lithiation cycle. We note that we do not consider small single-data-point fluctuations with high relevancy; we only consider clear trends and instances with the two measurement times. In the first cycle, no appreciable change in the Si peak position, ω_Si_, takes place between 2 and 1.5 V (the left-most part of Figure 5A, stage (0)). Starting at 1 V, the subsequent Si peak upshift by 0.6 cm^–1^ (from 516.6 to 517.2 cm^–1^ at 0.8 V) evidence a slight *c*-Si core compression (Figure 5D, stage (1, 2), yellow arrow) caused by the progressive growth of the bottom-SEI layer illustrated in Figure 5D as the olive shell (stage (1)), and top-SEI layer as a red shell (stage (2)), around the *c*-Si core. The local ω_Si_ maximum is perfectly aligned with the redox potential assigned to the SEI layer formation (0.8 V; cf. Figure 3A). Once the SEI layers are created, the following lithiation of *c*-Si causes tensile (Figure 5D, stage (3), green arrow) lattice deformation (down-shift of ω_Si_ with the minimum of 516.2 cm^–1^ at 0.1 V) of the *c*-Si core. This tensile deformation is associated with volume expansion at the reaction front that displaces the already lithiated phase,^60^ whereby the amorphous *a*-Li_3.5_Si phase is formed (Figure 5D, dark amber shell). The subsequent significant compressive deformation from 0.1 to 0.05 V is caused by the pressure imposed by the Li-rich shell (*c*-Li_3.75_Si layer, Figure 5D, stage (4), indigo shell) on the remaining nonlithiated *c*-Si core, as theoretically described by a model of concurrent reaction and plasticity by Zhao et al.^61^ The presence of the Li–Si alloy phases would be represented by the appearance of Raman bands in the spectral region below 450 cm^–1^.^62^ Nevertheless, due to the low intensity of these bands, we could not detect them during our in situ Raman SEC measurements (see Figure S2).

During the initial delithiation within the first cycle (right part of Figure 5A), the lattice recontraction of the *c*-Si core resulting from the shell delithiation takes place continuously from 0.05 to 0.3 V (from 516.9 to 517.7 cm^–1^) for holding time of 600 s (solid pink line with full circles in Figure 5A on the right) and up to 0.5 V (to 518.4 cm^–1^) for holding time 1200 s (dashed pink line with empty triangles), respectively. The longer delithiation time at 0.5 V causes an ongoing significant lattice contraction of the *c*-Si core, which is attributed to the more efficient phase transformation of *a*-Li_3.5_Si to *a*-Li_2_Si (Figure 5D, stage (5), light brown shell). Thereby, a thicker shell is created, which compresses the *c*-Si core. In contrast, the incomplete phase transformation during the shorter holding time results in an earlier onset of lattice relaxation in the *c*-Si core.^51^ The downshift of the *c*-Si Raman peak position from 0.3 V (from 0.5 V for 1200 s holding time) to 1 V can be explained by the complete dealloying linked with the creation of a thin *a*-Si layer (Figure 5D, stage (6), black shell) on the *c*-Si core. Whereas the Raman signature of the amorphous phase itself (a broad peak centered at ∼480 cm^–1^) is not discernible due to the overall low signal-to-noise ratio in the in situ spectra, its presence is evidenced by ex situ Raman measurements (Figure S5) after 2 cycles, where an obvious intensity decrease and asymmetry of the Si peak toward lower wavenumbers are clearly observed. Two possible reasons for the marked Raman peak downshift reaching 1 V can be considered: (i) the presence of *a-*Si shifting the spectral weight of the peak to lower wavenumbers, and (ii) relaxation of the *c*-Si core after its abrupt contraction. Option (i) is, however, less probable because of the significant FWHM drop accompanying the Raman peak downshift (Figure 5B). In addition, a very similar temporary lattice expansion was observed (although not commented upon) in a later stage of the delithiation process by Tardif et al.^35^ by operando XRD, which reflects only the changes in the *c*-Si lattice. However, it is important to note that the operando Raman spectroscopy in ref (35) does not show an analogous level of detail of observed structural changes compared to their synchrotron XRD investigation or the Raman spectroscopy in the present work. We can seek the explanation in several aspects of the used methodologies: different sensitivity of XRD and Raman spectroscopy, electrochemical procedure used for charging/discharging (galvanostatic chronopotentiometry with XRD in ref (35) vs cyclic voltammetry with Raman spectroscopy in ref (35) vs potential-step chronoamperometry in this work) and its duration, potential window, size of the Si particles (average 80 nm in ref (35) vs 6 nm in this work), and the electrolyte system (FEC additive in ref (35). After the phase transformations, a gradual relaxation of the *c*-Si core takes place up to the charging potential of 2 V, accompanied by a slight upshift of the Raman peak, probably caused by the shrinking of Si particles to their initial size.^63^

In the second cycle (blue lines in Figure 5), similar processes are observed; however, during lithiation in the SEI layer formation region, the *c*-Si core compression is not as pronounced as in the first cycle. This is also reflected in the almost complete disappearance of the reduction peak related to the growth of the top-SEI layer in cyclic voltammograms (Figure 3A, Section 3.2). The second delithiation (Figure 5A on the right, light blue line) mechanism is also similar to that of the first cycle, albeit with a noticeable difference. After the initial abrupt recompression, two *c*-Si core lattice fluctuations corresponding to phase transformations (*a*-Li_3.5_Si and *a*-Li_2_Si) are visible for both holding times at potentials akin to those of cyclic voltammetry peaks (0.3 and 0.5 V). Note that the light blue solid and dashed lines in Figure 5A overlap in this potential range. This indicates a time-independent effective delithiation process. The FWHM evolution in Figure 5B reflects the level of homogeneity and disorder of the *c*-Si lattice upon (de)lithiation. During the first lithiation cycle, after the lithiation of the surface oxide and SEI layers growth, the silicon nanoparticles become more homogeneous, followed by an increased heterogeneity and/or disorder caused by the delithiation process. This effect is then repeated in the second cycle.

As mentioned above, our in situ Raman SEC results do not show the transition of the crystalline silicon particles to a completely amorphous state of the Li–Si alloy (similarly to ref (35), where they observed gradual *c*-Si Raman peak intensity increase within delithiation), in contrast to other works.^31,32,34,64^ The continuous disappearance of the intensity of the *c*-Si Raman peak within refs^31,32,34,64^ may partially be caused by the progressive thickening of the SEI^23^ and *a*-Li_*x*_Si layers on the crystalline Si core absorbing some of the incident/scattered light.^36^ However, our in situ Raman results indicate that a considerable part of the Si particles’ volume still remains in the crystalline phase during the initial two cycles. This suggests the formation of relatively thin outer layers on our SiNPs during (de)lithiation, which allow for the observation of stress evolution in the unreacted *c*-Si core using in situ Raman SEC. This was also observed in an in situ TEM study,^60^ where the lithiated Li_*x*_Si shells crystallized into the Li_3.75_Si phase as lithiation proceeded while the Si cores were still present. We also note that Zeng et al.^36^ seemingly observed the opposite trend in tensile-compressive stress evolution during their in situ Raman measurements. However, their SEC measurement was performed during galvanostatic lithiation and with larger Si particles. Hence, it is possible that the formation of initial SEI layers was not captured in the Raman spectra. Following that, the results from their galvanostatic measurement are qualitatively similar to those presented in our work. A quick drop in discharge voltage corresponds to the SEI formation, but no *c*-Si lattice change was observed in the synchrotron XRD experiment of ref (35), using galvanostatic chronopotentiometry and larger Si particles. It should also be considered that the reaction may not be entirely homogeneous, meaning that there could be unreacted c-Si particles during the first cycles. However, in our case, the decreasing FWHM of the Si peak at the end of the lithiation suggests that the effect occurs uniformly across all Si particles, indicating a homogeneous lithiation process. In contrast, for a nonhomogeneous reaction where some particles would remain unreacted, the FWHM would increase, and the peak would become more asymmetric toward lower wavenumbers.

## 4 Conclusions

The evolution of stress in crystalline silicon nanoparticles (SiNPs) during the first two lithiation and delithiation cycles was examined by using in situ Raman spectroelectrochemistry (SEC). The experiments were conducted with noncommercial SiNPs synthesized by the PECVD method and exhibited a narrow and uniform particle size distribution with an approximate size of 6 nm. We observed real-time potential-dependent structural changes in SiNPs, as well as the formation of the solid electrolyte interphase (SEI) layer within the anode of lithium-ion batteries. Based on these results, we have proposed a qualitative model elucidating the structural changes within the *c*-Si core of SiNPs and their dependence on the applied lithiation/delithiation potential. The cycling stability measurements of the silicon anode showed a reversible capacity of approximately 650 mAh/g after 100 galvanostatic charge/discharge cycles. The observed intensity and shifts of the *c*-Si Raman band exhibited distinct behaviors during the first and second lithiation cycles. During the first cycle, the intensity drop was more pronounced: upon lithiation, the Si peak intensity remained relatively stable until 0.3 V, followed by a rapid decrease until the delithiation potential reached 0.3 V again, with a subsequent gradual increase in intensity until the end of delithiation. In the second cycle, the Si peak intensity displayed greater stability with only minor fluctuations observed at lower potentials. The observed intensity decrease of the electrolyte Raman bands during the first lithiation cycle suggests its decomposition and SEI layer formation (at potentials up to 0.8 V), while the less varying intensities of these bands during the second cycle indicate SEI layer stabilization. The changes (shifts) in the Si Raman peak position indicate lattice deformation within the *c*-Si. During the first lithiation, *c*-Si core compression (Raman peak upshift) occurs due to SEI layer formation, followed by tensile (downshift) stress as the lithiation continues. The formation of the amorphous *a*-Li_*x*_Si phase during lithiation was accompanied by volume expansion and the subsequent compressive deformation induced by pressure from the Li-rich shell on the remaining nonlithiated *c*-Si core. Delithiation within the first cycle involved a lattice recontraction of the *c*-Si core, followed by complete dealloying (downshift) and the formation of a thin *a*-Si layer. In the second cycle, similar processes occurred, with a less pronounced *c*-Si core compression during lithiation and a notable reduction in the cyclic voltammetry peak related to the top-SEI layer growth. Delithiation mechanisms mirrored those of the first cycle, with distinct lattice fluctuations indicating time-independent effective delithiation. The Raman line width evolution demonstrated increased heterogeneity/disorder of the *c*-Si lattice during delithiation within both cycles.

## Supporting Information Available

Figure S1: Long-term Raman measurements before in situ Raman SEC of the SiNP@CB, Figure S2: in situ Raman-SEC spectra (experimental) of the SiNP@CB in the whole region (200–1000 cm^–1^) of Si and electrolyte vibrations, and details of the individual spectral regions; (A) *c*-Si region (480–560 cm^–1^) and electrolyte region, (B) 700–760 cm^–1^, (C) 870–940 cm^–1^, during the 1st and 2nd (de)lithiation cycles, Figure S3: (A) in situ Raman-SEC spectra (Lorentzian fit) of the SiNP@CB in the region of Si vibrations (490–550 cm^–1^) during the first and second (de)lithiation cycles and (B) intensity of the Si Raman peak, Figure S4: in situ Raman-SEC spectra (Lorentzian fit) of the SiNP@CB in the region of electrolyte vibrations (A, C in 700–760 cm^–1^ region; and B, D in 870–940 cm^–1^ region) during the first and second (de)lithiation cycle, and Figure S5: ex situ Raman spectra of pristine SiNP@CB electrode and electrode after the second cycle (PDF)
