# Supplementary material for: Structural and Chemical Changes in Si Nanoparticle-Based Anodes in Lithium-Ion Batteries during the (De)lithiation Processes Studied by In Situ Raman Spectroelectrochemistry
Source: ACS Appl Energy Mater. 2025 Apr 28;8(9):5729–37. doi: 10.1021/acsaem.5c00066 (PMC12076281; doi:10.1021/acsaem.5c00066)
Supplement: Supplementary file 1 — ae5c00066_si_001.pdf [file ae5c00066_si_001.pdf]

Supporting Information

Structural and chemical changes in Si nanoparticle-based anodes in lithium-ion batteries during the (de)lithiation processes studied by *in situ* Raman spectroelectrochemistry

Zuzana Vlčková Živcová[1], Farjana J. Sonia[1,†], Martin Jindra[1,2], Martin Müller[3,‡], Jiří Červenka[3], Antonín Fejfar[3] and Otakar Frank[1]*

[1] J. Heyrovský Institute of Physical Chemistry, Czech Academy of Sciences, 182 23 Prague, Czech Republic

[2] Department of Physical Chemistry, University of Chemistry and Technology, 16628 Prague, Czech Republic

[3] Institute of Physics of the Czech Academy of Sciences, 182 21 Prague, Czech Republic

[†] Current affiliation: Institute for Metallic Materials, Leibniz Institute for Solid State and Materials Research Dresden, 01069 Dresden, Germany

[‡] Current affiliation: Institute for Materials Science, Synthesis and Real Structure, Kiel University, Kaiserstr. 2, 24143 Kiel, Germany

*email: zuzana.vlckova@jh-inst.cas.cz

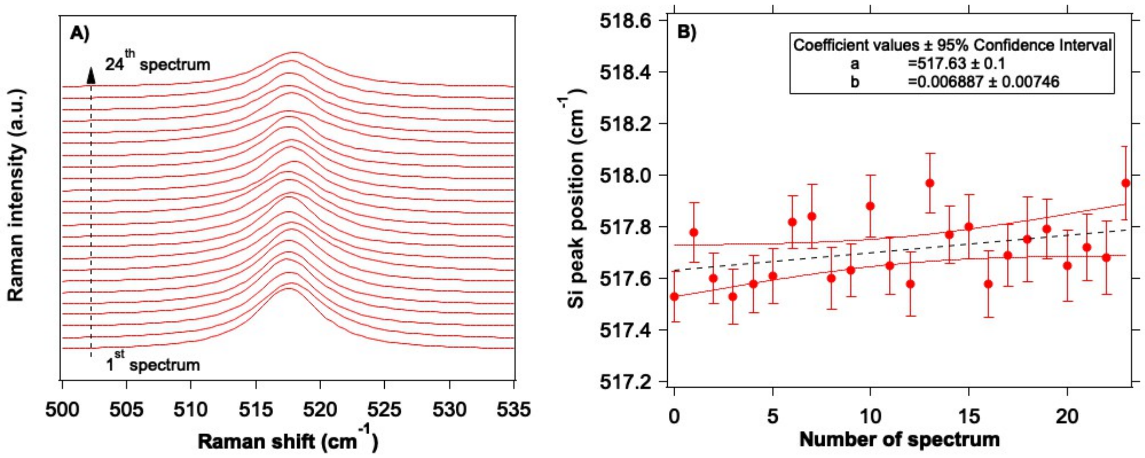

Figure S1. A) Lorentzian fit of *in situ* Raman spectra of the Si Raman peak of the SiNP@CB pristine electrode without applied potential (no electrical contact) in the 480–540 cm^{-1} region, and B) Si Raman peak position determined from the Lorentzian fits. The spectra were excited by 633 nm laser radiation and recorded during 40 minutes, offset for clarity; the intensity scale is identical for all the curves. The diffraction grating was 1800 lines/mm.

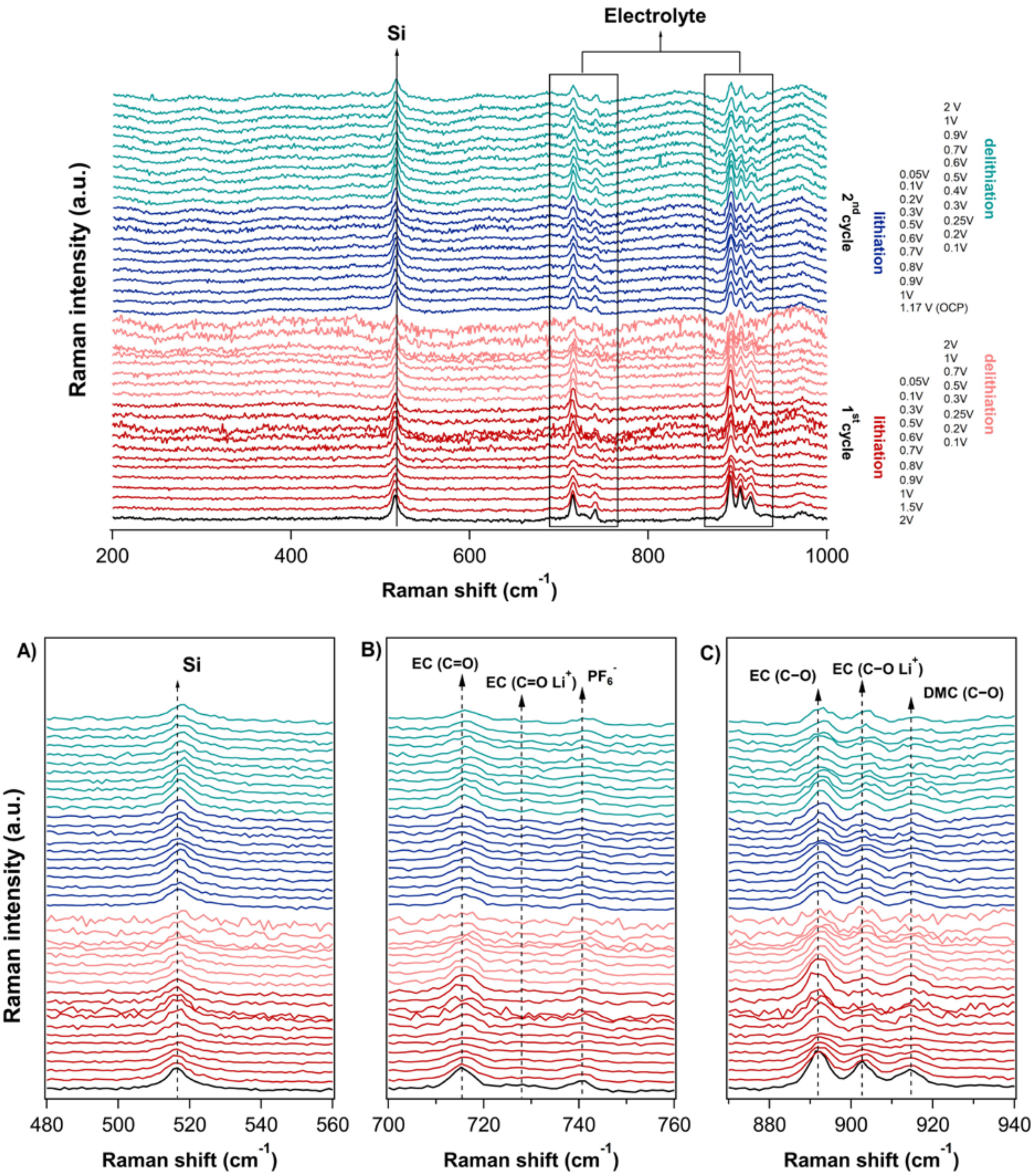

Figure S2. The set of *in situ* Raman SEC spectra (top chart) of the SiNP@CB electrode as a function of the applied potential (vs. Li/Li^+) in the 200–1000 cm^{-1} spectral region, and a detail of the individual spectral regions; A) *c*-Si region, and B), C) electrolyte regions, used for fitting in Figures S3A and Figure S4. The measurement sequence is as follows; the 1st cycle is shown in purple traces (the dark trace for lithiation from 2 V to 0.05 V, and the light trace for delithiation from 0.1 V to 2 V), and the 2nd cycle is shown in blue traces (the dark trace for lithiation from OCP of 1.17 V to 0.05 V, and the light trace for delithiation from 0.1 V to 2 V). The reference Raman spectrum of the pristine electrode before cycling is shown for comparison (bottom black trace). The spectra were excited by 633 nm laser radiation and offset for clarity. All spectra are recorded at a fixed potential at the holding time t = 600 s using diffraction grating of 600 lines/mm.

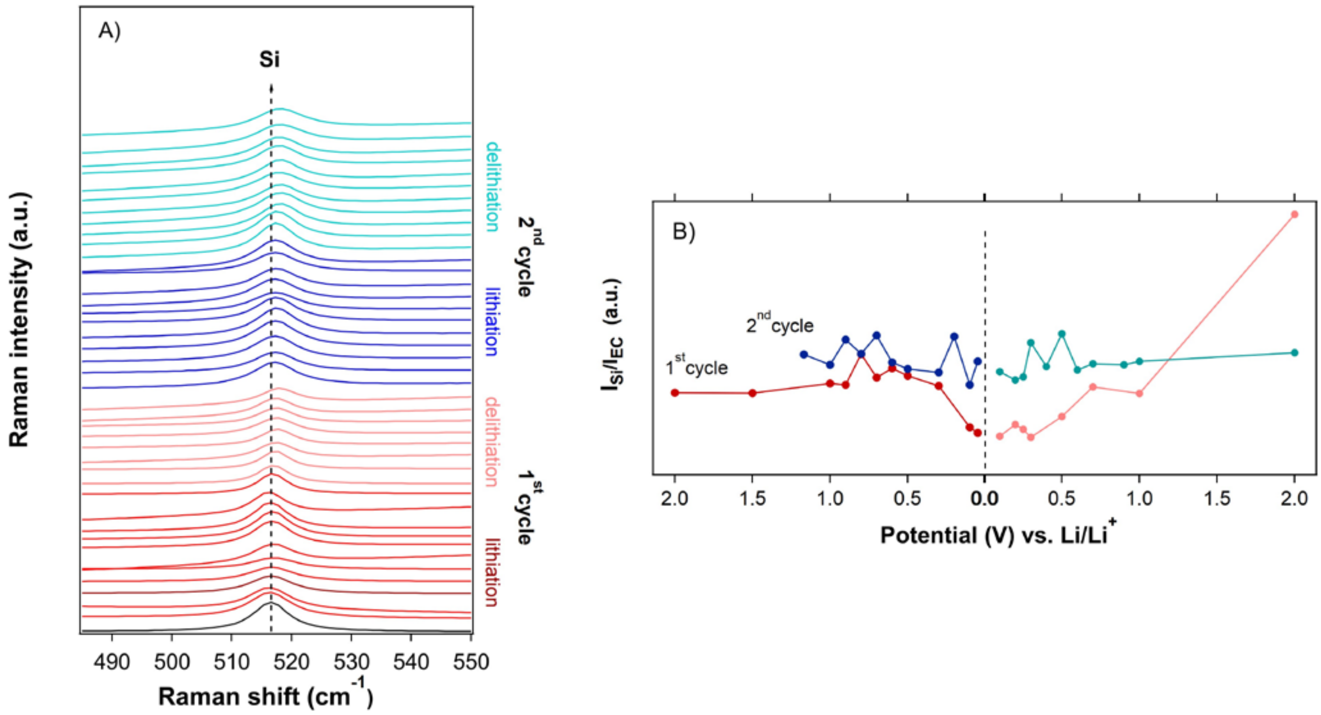

Figure S3. A) The Lorentzian fit of *in situ* Raman SEC spectra from Figure S2 of the Si Raman peak in the 480–540 cm^{-1} region, and B) the intensity of the Si Raman peak at ~520 cm^{-1} normalized to the intensity of the EC (C=O) electrolyte Raman peak at 715 cm^{-1}. All spectra were recorded at a fixed potential at t = 600 s.

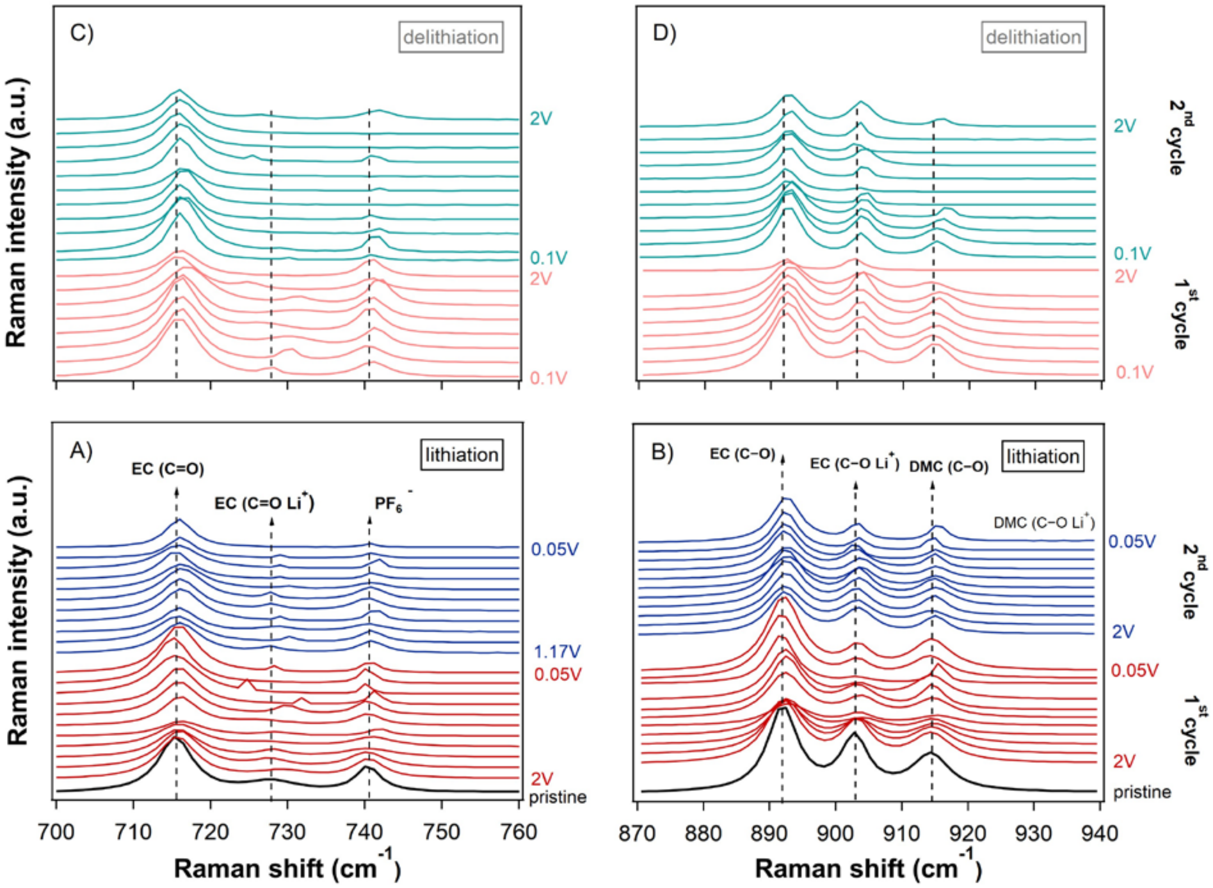

Figure S4. The sum of Lorentzian fits of individual Raman peaks of the electrolyte solution ($LiPF_6$ in EC/DMC) from *in situ* Raman SEC spectra from Figure S2 as a function of the applied potential (vs. Li/Li^+) in the 700–760 cm^{-1} region (A - lithiation, C - delithiation) and 870–940 cm^{-1} region (B - lithiation, D - delithiation) within 1st cycle (pink traces) and 2nd cycle (green traces). The reference Raman spectrum of the electrolyte before cycling is shown for comparison (bottom black trace in A and B). The diffraction grating was 600 lines/mm. All spectra were recorded at a fixed potential at t = 600 s.

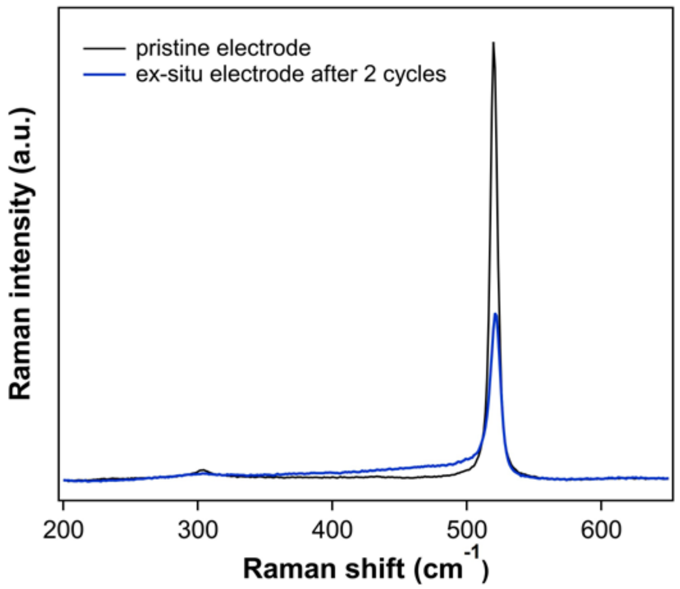

Figure S5. *Ex situ* Raman spectra of the SiNP@CB pristine (black line) electrode and the electrode after 2 cycles (blue line). The spectra were excited by 633 nm laser radiation. The diffraction grating was 600 lines/mm.
